# Environmental Niche Modelling of Phlebotomine Sand Flies and Cutaneous Leishmaniasis Identifies *Lutzomyia intermedia* as the Main Vector Species in Southeastern Brazil

**DOI:** 10.1371/journal.pone.0164580

**Published:** 2016-10-26

**Authors:** Viviane Coutinho Meneguzzi, Claudiney Biral dos Santos, Gustavo Rocha Leite, Blima Fux, Aloísio Falqueto

**Affiliations:** 1 Tropical Medicine Unit, Department of Pathology, Federal University of Espírito Santo (Universidade Federal do Espírito Santo), Vitória, ES, Brazil; 2 Nucleus of Entomology and Malacology (Núcleo de Entomologia e Malacologia) of the Espirito Santo State Health Secretariat, Serra, ES, Brazil; 3 Tropical Medicine Unit, Department of Social Medicine, Federal University of Espírito Santo (Universidade Federal do Espírito Santo), Vitória, ES, Brazil; Quensland University of Technology, AUSTRALIA

## Abstract

Cutaneous leishmaniasis (CL) is caused by a protozoan of the genus *Leishmania* and is transmitted by sand flies. The state of Espírito Santo (ES), an endemic area in southeast Brazil, has shown a considerably high prevalence in recent decades. Environmental niche modelling (ENM) is a useful tool for predicting potential disease risk. In this study, ENM was applied to sand fly species and CL cases in ES to identify the principal vector and risk areas of the disease. Sand flies were collected in 466 rural localities between 1997 and 2013 using active and passive capture. Insects were identified to the species level, and the localities were georeferenced. Twenty-one bioclimatic variables were selected from WorldClim. Maxent was used to construct models projecting the potential distribution for five *Lutzomyia* species and CL cases. ENMTools was used to overlap the species and the CL case models. The Kruskal–Wallis test was performed, adopting a 5% significance level. Approximately 250,000 specimens were captured, belonging to 43 species. The area under the curve (AUC) was considered acceptable for all models. The slope was considered relevant to the construction of the models for all the species identified. The overlay test identified *Lutzomyia intermedia* as the main vector of CL in southeast Brazil. ENM tools enable an analysis of the association among environmental variables, vector distributions and CL cases, which can be used to support epidemiologic and entomological vigilance actions to control the expansion of CL in vulnerable areas.

## Introduction

Cutaneous leishmaniasis (CL) is a disease caused by protozoans of the genus *Leishmania* Ross, 1903 and is transmitted by insects from the subfamily Phlebotominae [1–2]. Decades ago, CL was exclusively presented as an enzootic disease of wild animals that occasionally affected humans in forest habitats. The disease has been considered an emerging infectious disease, and an increased prevalence has been observed, which is associated with deforestation, exploitation of natural resources, agricultural expansion, and migration of populations [3].

Currently, the disease occurs in deforested rural and peri-urban areas in Brazil [4–6]. In these regions, both adults and children are living with the disease, and domestic dogs have been suggested to be an important source of infection [7–9].

CL is well distributed throughout Brazil, including the state of Espírito Santo (ES), located in the southeast, where it is widely distributed and affects predominantly rural populations [10–12]. Until the mid-1980s, the disease was predominant in western ES, where the geo-climatic conditions presumably favoured the development of both the vector and the wild reservoirs of the disease, which are grouped mainly in the Rodentia order (e.g., *Akodon* sp.) [12,13]. After the mid-1980s, however, changes in the economic structure of the state caused changes in the population density profile, which allowed the geographic expansion of CL towards the eastern ES, most likely associated with domestic reservoirs such as dogs and horses [12–14]. In these areas, the increased population density in the periphery of urban centres favoured the displacement of domestic reservoirs through houses, increasing the incidence of CL [8,10].

Approximately 800 species of phlebotomine sand flies have been described worldwide, and approximately 60% of them occur in the Neotropics [1–2]. In Brazil, more than 260 species of phlebotomine sand flies have been recorded. They are distributed in all geographical regions [15], but the majority of them occur in the Amazon basin and in northeast Brazil.

In ES, approximately 59 species of sand flies have been recorded, among which, three species likely have epidemiological importance in disease transmission: *Lutzomyia intermedia* (Lutz & Neiva, 1912), *Lutzomyia migonei* (França, 1920), and *Lutzomyia whitmani* (Antunes & Coutinho, 1939) [16,17–20]. Other common species, such as *Lutzomyia choti* (Floch and Abonnenc, 1941) and *Lutzomyia lenti* (Mangabeira, 1938), seem to not be associated with the disease occurrence in ES [5]. However, no consistent investigation has been conducted on the role of these species in the disease transmission, its animal reservoirs, and the interactions between vector species and environmental conditions [21].

The use of the environmental approach offered by landscape epidemiology allows us to investigate the environmental aspects associated with the disease [22]. Recently, sophisticated techniques, such as environmental niche modelling (ENM), have been used to better understand the potential distribution of the species involved in the disease transmission, as well as to predict areas of potential risk.

Accordingly, the present study aimed to use spatial modelling techniques to identify vector species and their potential distribution and relationship with the occurrence of CL in ES, southeastern Brazil.

## Materials and Methods

### Study area

**Fig 1:**
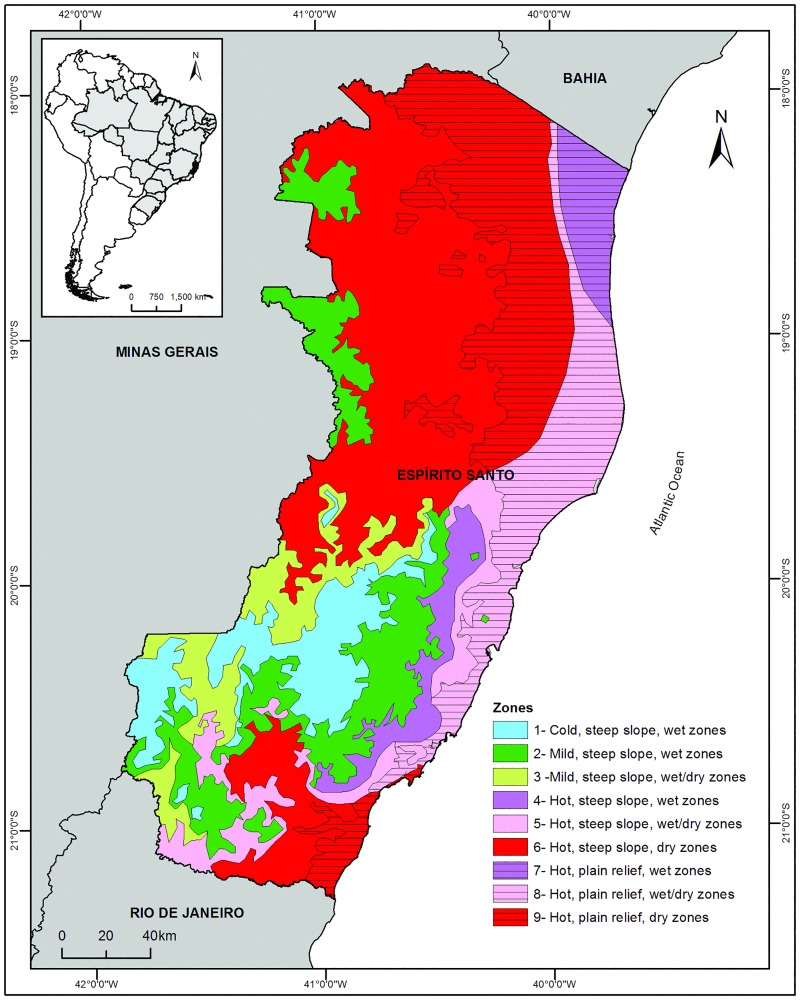
Geographical location of the state of Espírito Santo, southeastern Brazil, South America and the geo-climatic zones. By definition, a hot zone has a minimum average temperature of 11.8–18.0°C and a maximum of 30.7–34.0°C. A mild zone has a minimum average temperature of 9.4–11.8°C and a maximum of 27.8–30.7°C. A cold zone has a minimum average temperature of 7.3–9.4°C and a maximum of 25.3–27.8°C. A steep slope zone has a slope above 8%, and a plain relief occurs when the slope is below 8%. A wet zone has < 4 dry months per year, a wet/dry zone has 4–6 dry months, and a dry zone has > 6 dry months [21]. Datum: SIRGAS 2000.

The state of ES is located on the Atlantic coast of southeastern Brazil, between longitudes 17°53'29''S 21°18'03''S and latitudes 39°41'18''W 41°52'45''W, with a geographical area of 46,096 km^2^ and different ecologic characteristics, according to geo-climatic zones (Fig 1) [23].

The annual average temperature of this area is 23°C, and the annual rainfall is above 1,400 mm. The elevation in some regions can reach 2,800 m above sea level [24]. This state forms the southern part of the Central Atlantic Forest Corridor, which is one of the main stretches of the dense ombrophilous forest found in this biome, presenting a high degree of endemism and species diversity [25].

### Entomological and epidemiological data

The occurrence data of phlebotomine sand fly species were obtained from samples taken in several rural areas of 78 municipalities of ES every year between 1997 and 2013. Each collection occurred during the first 3 hours after sunset through active and passive capture using a manual Castro-type suction tube and Centers for Disease Control and Prevention CO2 trap (CDC) and Shannon light traps installed in the areas surrounding homes. During active surveillance, insects were collected from the internal and external walls of homes, home additions, animal shelters, and tree trunks. Sampling was performed by well-trained technicians, regardless of the occurrence of disease outbreak. The species identification and nomenclature were in accordance with Young and Duncan [26].

The localities and epidemiological data of the CL cases in ES were extracted from the medical records of patients cared for at Hospital Universitário Cassiano Antônio de Moraes (HUCAM) from 1978—when an effort began to initiate a careful and accurate identification of infection, with characteristics being detailed—to 2013. HUCAM is a reference hospital used in CL diagnosis and treatment for the municipalities of ES, centralizing every patient attendance, which verifies that the sample is representative of CL occurrence in that region. The type of information extracted from the medical records included geographical coordinates of the locality where the patient was infected.

### Environmental analysis

Environmental variables from the WorldClim database were used for the modelling, with an accuracy to within approximately 1 km [24]. This database comprises 19 bioclimatic variables derived from temperature and precipitation, as well as terrain elevation, and calculated the percent slope that is a measure of terrain inclination relative to the horizontal plan.

These WorldClim variables (https://www.worldclim.org) include the annual mean temperature (BIO1), mean diurnal range (BIO2), isothermality (BIO3), temperature seasonality (BIO4—standard-deviation*100), maximum temperature of the warmest month (BIO5), minimum temperature of the coldest month (BIO6), temperature annual range (BIO7), mean temperature of the wettest quarter (BIO8), mean temperature of the driest quarter (BIO9), mean temperature of the warmest quarter (BIO10), mean temperature of the coldest quarter (BIO11), annual precipitation (BIO12), precipitation of the wettest month (BIO13), precipitation of the driest month (BIO14), precipitation seasonality (BIO15—coefficient of variation), precipitation of the wettest quarter (BIO16), precipitation of the driest quarter (BIO17), precipitation of the warmest quarter (BIO18), and precipitation of the coldest quarter (BIO19) [24].

The data for these bioclimatic variables were generated through interpolations of climate information from 1950 to 2000, obtained from approximately 50,000 weather stations distributed around the world [24]. The elevation data were obtained from the Shuttle Radar Topography Mission (SRTM) of the National Aeronautics and Space Administration (NASA). All variables had a 30-arc second (~1 km at the Equator) spatial resolution [24].

Spatial procedures of this study were performed using the Geographic Information System (GIS) ArcGIS ver. 10.1 (ESRI, Redlands, CA, USA) with Geocentric Reference System for the Americas (SIRGAS, 2000) data. All studied locations were georeferenced with a Global Positioning System (GPS).

### Environmental niche model of Phlebotominae species and CL

A maximum-entropy modelling approach using Maxent version 3.3.3k (https://www.cs.princeton.edu/~schapire/maxent/) was used to build potential distribution models of the five most common species, namely, *L*. *intermedia*, *L*. *migonei*, *L*. *whitmani*, *L*. *choti*, and *L*. *lenti* and the distribution of CL cases. Maxent is an a-based model from presence-background data that estimates a target probability distribution by finding the probability of distribution of maximum entropy. Maxent generates a “background” or “pseudo-absence” sample of the occurrence points. By default, 10,000 pseudo-absences are randomly selected from the entire rectangular study area [27]. Recently, this method has been widely used in species conservation studies. Similarly, these tools have been used in healthcare studies, presenting considerable development over the last 10 years [28–38].

The occurrence data for the species were separated into two sets, as follows: one set for the model calibration/training (75% of the occurrence localities) and the other set for the model evaluation/test (25% of the occurrence localities). The percentage contribution of each variable to the final model based on how much the variable contributed to the model dependent upon the path selected for a particular model run was provided by Maxent; in this heuristic approach, the contribution values are determined by the increase in gain in the model provided by each variable [27,39,40]. The model training and test procedure were replicated 10 times for each species, and the mean was calculated. The sampling technique used was Bootstrap, in which the training data are selected by sampling with replacement from the presence points, with the number of samples equalling the total number of presence points [40].

ENMtools version 1.3.3 (http://enmtools.blogspot.com.br/) was used to evaluate which species among those analysed in this study are more associated with the occurrence of the disease by overlapping the distribution of the vectors with the distribution of the CL cases. ENMtools overlaps the number of replicas generated by the Maxent software between individual species and cases of the disease using Schoener’s D index calculation, which evaluates the similarity between these models. Schoener’s D index was calculated with the construction of 10 replicates for each evaluated model, and therefore, 100 values of this index were obtained by analysing the overlap. The mean of this index was also compared to assess which species present a greater degree of niche overlap within the area of CL occurrence in the state of ES [41].

### Statistical analysis

The validation of the models was performed using the receiver operating characteristic (ROC) curve, in which the true positive fractions are plotted against the false positive fractions. The area under the curve (AUC) is used as a measure of accuracy of the model [39,42].

The AUC values range from 0.5 to 1.0. The classification system for the AUC provided by Hosmer and Lemeshow was used in the current study. That system classifies the AUC values as follows: 0.5–0.6 = no discrimination, 0.6–0.7 = poor discrimination, 0.7–0.8 = acceptable discrimination, 0.8–0.9 = excellent discrimination, and 0.9–1.0 = outstanding discrimination [43].

The Kolmogorov-Smirnov test was performed to determine the normality for Schoener’s D data and the variables’ percentage contribution distribution. The Kruskal-Wallis test with post-hoc Dunn’s test was used to compare the medians of this indicator between species. The Kruskal-Wallis test followed by the Student-Newman-Keuls test was used to compare the percentage contribution of the five most important variables in the model construction. The significance level adopted was 5%.

### Ethics statements

The study was approved by the Human Research Ethics Committee of the Health Sciences Centre of the Federal University of Espirito Santo (UFES) (opinion no. 494.029 from 13 December 2013). Patient information was anonymized and de-identified prior to analysis. The insects were collected on private lands with the permission of the landowners. No endangered species or protected areas were involved.

## Results

### Descriptive analysis

**Fig 2:**
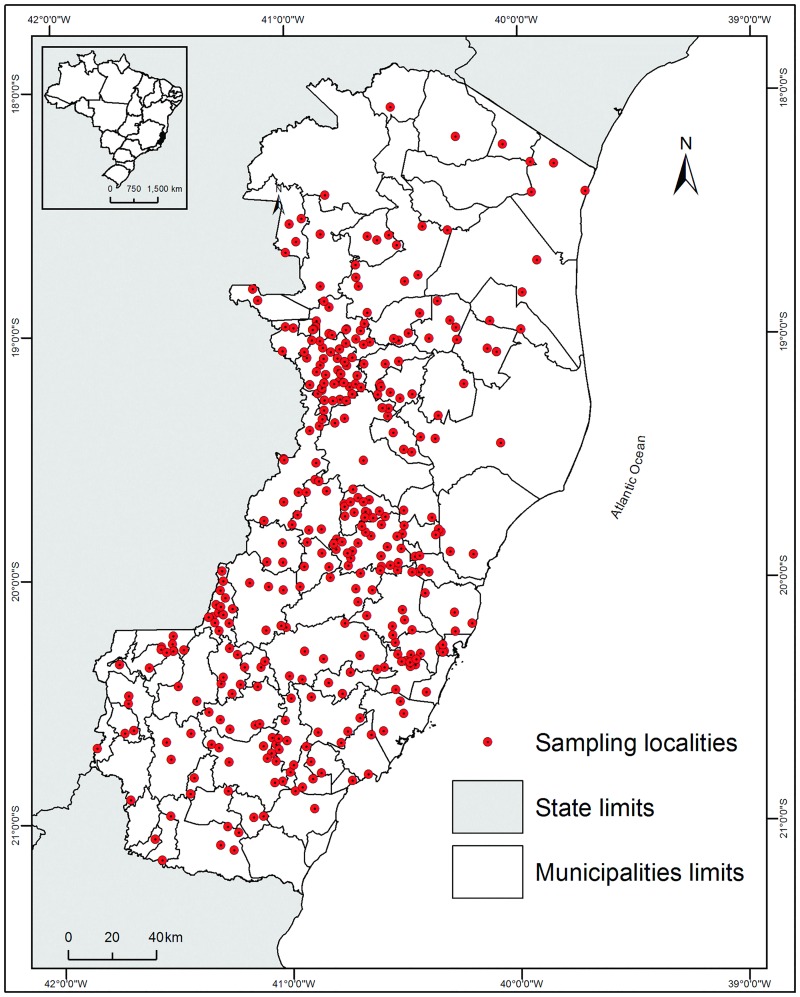
Sampling localities of phlebotomine sand flies in rural locations in the state of Espírito Santo, southeastern Brazil, during the period between 1997 and 2013. Datum: SIRGAS 2000.

In total, 249,783 specimens belonging to 43 species of phlebotomine sand flies were collected at 466 localities during the period between 1997 and 2013, covering all geo-climatic zones of the state of ES (Fig 2, S1 Table) [23].

### ENM of the main species of phlebotomine sand flies

**Fig 3:**
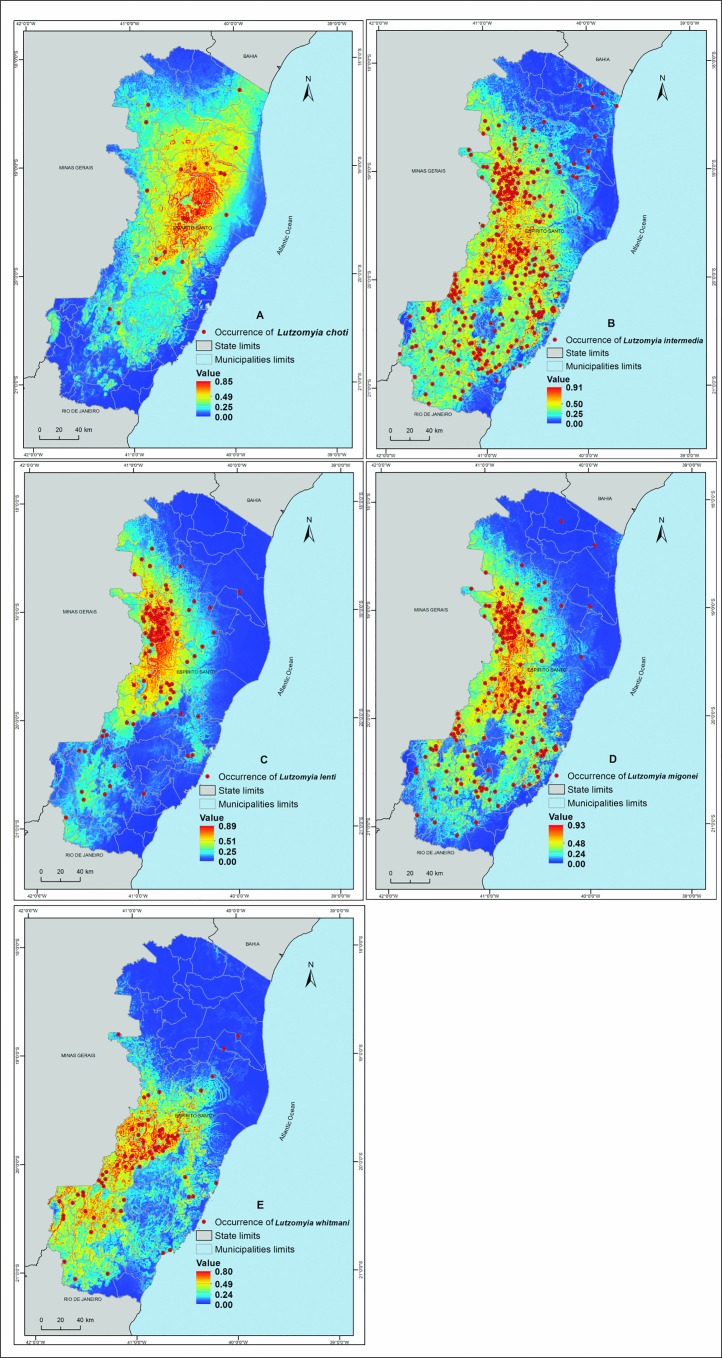
Predicted ecological niche distribution of five most frequent Phlebotominae species in the state of Espírito Santo, southeastern Brazil, during the period between 1997 and 2013. (A) *Lutzomyia choti*, (B) *Lutzomyia intermedia*, (C) *Lutzomyia lenti*, (D) *Lutzomyia migonei* and (E) *Lutzomyia whitmani*. The species occurrence probability is expressed with values ranging from 0 to 1. Datum: SIRGAS 2000.

For the construction of the environmental niche models of the main vectors of the disease, the five species of phlebotomine sand flies most commonly reported in the state of ES were selected: *L*. *intermedia*, *L*. *migonei*, *L*. *choti*, *L*. *lenti*, and *L*. whitmani, excluding *L*. *longipalpis*, which is a vector of visceral leishmaniasis (Fig 3).

The AUCs were greater than 0.80, indicating excellent discrimination for *L*. *migonei*, *L*. *choti*, *L*. *lenti*, and *L*. *whitmani* (Table 1). The model was also considered acceptable for *L*. *intermedia*; however, the average AUC was lower than the averages of the other species (0.78).

**Table 1:** Number of occurrence localities of phlebotomine sand fly species collected in rural locations in the state of Espírito Santo. The mean value and standard deviation (sd) for the AUC are reported.

Variable	Number of occurrences	AUC mean (sd)
*Lutzomyia intermedia*	404	0.780 (0.025)
*Lutzomyia migonei*	255	0.837 (0.027)
*Lutzomyia choti*	24	0.824 (0.078)
*Lutzomyia lenti*	123	0.857 (0.033)
*Lutzomyia whitmani*	68	0.815 (0.053)

According to the analysis of the variables using the percent contribution heuristic test, all species showed high sensitivity to temperature, precipitation, elevation, and slope. Therefore, slope was an important predictor and contributed 37.85%, 33.62%, 20.32%, 15.61%, and 11.28% for the species *L*. *intermedia*, *L*. *whitmani*, *L*. *migonei*, *L*. *choti*, and *L*. *lenti*, respectively (S2 Table).

Regarding the other variables, *L*. *intermedia* and *L*. *migonei* showed similarities in the construction of their models. Slope, BIO13, BIO12, BIO5, and elevation contributed 71.71% to the construction of the model for *L*. *intermedia*, whereas slope, BIO13, BIO16, elevation, and BIO12 contributed 59.31% to the construction of the model for *L*. *migonei*.

*Lutzomyia lenti* and *L*. *whitmani* also presented similarities. BIO15, slope, BIO13, BIO16, and BIO11 contributed 69.27% to the construction of the model for *L*. *lenti*, whereas slope, BIO4, BIO13, BIO15, and BIO5 contributed 79.36% to the construction of the model for *L*. *whitmani*. Except for slope, *L*. *choti* did not present similarities with the other species. Slope, BIO4, BIO18, elevation, and BIO17 contributed 66.05% to the construction of the model for this species (Table 2).

**Table 2:** Environmental variable values of the sample sites of five phlebotomine species in the state of Espírito Santo, Brazil, 1997–2013. The mean value and standard deviation (sd) for each variable are reported.

Species	Environmental variables						
	Elevation (m)	Slope (%)	BIO4 (sd)	BIO5 (°C)	BIO12 (mm)	BIO13 (mm)	BIO15 (cv^a^)
***Lutzomyia intermedia (*mean ± sd)**	333.10 (271.74)	6.57 (5.12)	16.62 (1.25)	30.92 (1.46)	1190.12 (65.61)	198.50 (12.18)	59.00 (6.39)
***Lutzomyia migonei (*mean ± sd)**	346.15 (267.76)	6.67 (4.81)	16.55 (1.22)	30.91 (1.46)	1192.39 (62.79)	199.57 (11.31)	59.73 (6.14)
***Lutzomyia whitmani (*mean ± sd)**	443.53 (310.76)	7.84 (4.96)	17.51 (1.04)	30.52 (1.65)	1217.40 (71.80)	206.38 (14.18)	59.93 (5.88)
***Lutzomyia choti (*mean ± sd)**	252.67 (726.16)	5.62 (14.95)	16.03 (0.02)	30.93 (9.65)	1197.38 (15.59)	192.00 (54.78)	55.25 (7.17)
***Lutzomyia lenti (*mean ± sd)**	265.58 (188.90)	5.95 (4.77)	16.05 (1.12)	31.42 (1.00)	1174.86 (43.97)	197.93 (8.97)	62.11 (4.06)

^a^cv, coefficient of variation.

### ENM of CL occurrence area

**Fig 4:**
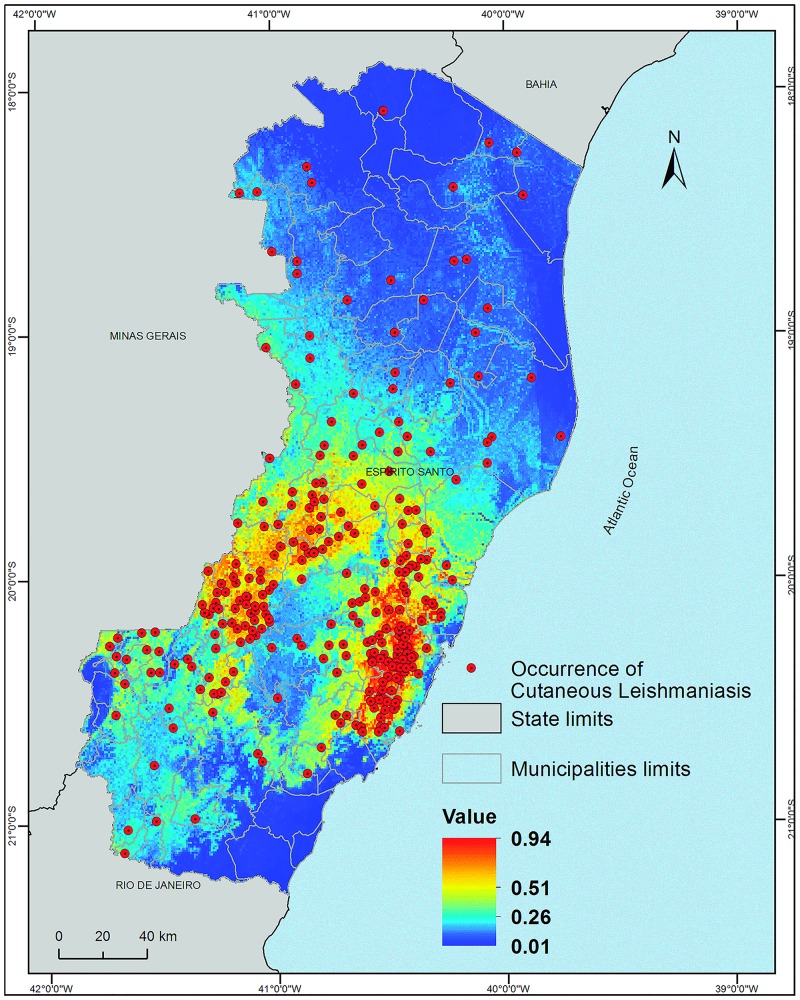
Predicted distribution of the ecological niche of the area of occurrence of autochthonous human cases of cutaneous leishmaniasis in the state of Espírito Santo, Brazil, 1978–2013. Datum: SIRGAS 2000.

In total, 1,472 autochthonous human cases of CL were reported between 1978 and 2013. Among these cases, 1,264 (85.9%) presented cutaneous lesions, 49 (3.3%) presented mucocutaneous lesions, and 159 (10.8%) presented mucosal lesions. The model constructed in the current study shows that this disease occurs in the south-central region of the state of ES and is limited to areas below 1,119 metres; however, only 17 (4.76%) of 357 occurrence localities were related in elevation above 850 metres (Fig 4).

The model was considered excellent, with an AUC of 0.817 ± 0.020. The variables BIO4, BIO12, slope, BIO14, and BIO5 contributed 70.31% of the model construction (Table 3, S2 Table).

**Table 3:** Mean and standard deviation of the most relevant environmental variables of the occurrence of cutaneous leishmaniasis in the state of Espírito Santo, Brazil, 1978–2013.

Variable	Elevation (m)	Slope (%)	BIO4 (sd)	BIO5 (°C)	BIO12 (mm)	BIO13 (mm)	BIO15 (cv^a^**)**
Mean (sd)	317.80 (281.81)	6.41 (5.15)	17.13 (0.91)	30.84 (1.37)	1188.53 (62.04)	197.36 (13.10)	54.47 (7.62)
Upper bound	1,119.00	30.85	20.05	33.50	1375.00	234.00	70.00
Lower bound	1.00	0.04	14.81	26.20	1026.00	166.00	38.00

^a^cv, coefficient of variation.

### Overlap between the distribution of human CL cases and Phlebotominae species

Values of Schoener's D range from 0 (no overlap) to 1 (perfect overlap). This indicator was compared between species to evaluate which one is most involved in disease transmission (Table 4). The Kruskal-Wallis and Dunn tests (multiple comparisons) were used to define the importance of each species. In the state of ES, the *L*. *intermedia* model showed significantly higher overlap, indicating that it is more associated with the disease transmission than secondary vectors such as *L*. *migonei* and *L*. *whitmani* (p < 0.05), with no difference between the latter two species (p > 0.05), followed by *L*. *lenti* and *L*. *choti*, most likely without epidemiological importance in the transmission.

**Table 4:** Schoener’s D mean and standard deviation for the overlap between 10 replicas of the models of Phlebotominae species and 10 replicas of the cutaneous leishmaniasis model.

Phlebotominae species	Schoener’s D*
*Lutzomyia intermedia*	0.725 (0.019)^a^
*Lutzomyia migonei*	0.688 (0.023)^b^
*Lutzomyia whitmani*	0.675 (0.044)^b^
*Lutzomyia lenti*	0.582 (0.050)^c^
*Lutzomyia choti*	0.561 (0.055)^c^

* Different letters indicate statistically significant differences between groups.

## Discussion

This is the first study to build and associate environmental niche models using algorithms between vector species and autochthonous CL cases in endemic areas of south-eastern Brazil.

Environmental modelling research focused on public health is under considerable development in Brazil and worldwide. In Portugal, Spain, France, and Italy, studies have mapped the environmental risk of visceral leishmaniasis, using the potential distribution of canine leishmaniasis and their vectors [28,29,30]. In North America, the risk of leishmaniasis from the ENM of vectors and reservoirs has been evaluated to explain the expansion of the disease in Mexico [31]. In Colombia, South America, future projections of the *Leishmania infantum* (Nicole, 1908) spatial distribution have been studied [32]. Aparicio and Bitencourt modelled the risk areas for CL in the municipality of Itapira, São Paulo [34]. Almeida et al. evaluated the potential distribution of *Lutzomyia longipalpis* and cases of visceral leishmaniasis in Mato Grosso do Sul [35]. Nieto and collaborators used the modelling to define environmental niches of visceral leishmaniasis in Bahia [36]. Peterson and Shaw investigated niche models and effects of climate change in vectors of CL distribution in southeastern Brazil [37]. Almeida and Werneck evaluated predictive areas of high risk for visceral leishmaniasis in the city of Teresina, Piauí [38].

The current study revealed that *L*. *intermedia* was the species most associated with the occurrence of CL in the state of ES, followed by the secondary vectors *L*. *migonei* and *L*. *whitmani*. However, the species *L*. *lenti* and *L*. *choti* were not considered important vectors of the disease in that region.

*Lutzomyia intermedia* has been indicated as the main vector of CL in the state of ES [9,10]. This species is abundant in endemic areas of southeastern Brazil, and specimens have been naturally infected by *Leishmania* (*Viannia*) *braziliensis* Vianna, 1911 [5,11,44–46]. This species is more associated with domiciles and peridomiciles and is strongly attracted to domestic dogs and horses [8–9,10,47]. Studies suggest that this species is adapted to habitats modified by human activity and presents a high degree of anthropophily and endophily [45,47].

*Lutzomyia migonei* has been considered a secondary vector of CL in anthropic environments, with occurrence at altitudes above 750 m in ES [11]. This species has an affinity for humans and domestic dogs. A study conducted in the municipality of Afonso Cláudio (mid-western region of the state) revealed that *L*. *migonei* is the most common species found inside the domicile [45,47–48].

*Lutzomyia whitmani* has also been considered a secondary vector of CL, with high density in the endemic areas of the states of São Paulo, Minas Gerais, and ES [11,49–50]. This species is more abundant in protected forest areas. Recent studies have identified this species in peridomiciliary areas, especially in domestic animal shelters [9,11,45,47]. This species has been implicated as being responsible for the link between the wild environment and areas surrounding homes; however, it has a low propensity to invade households.

Rocha et al. [51] suggested that a greater genetic diversity of *Leishmania braziliensis* is observed in areas of remaining forests due to the larger number of reservoirs and vector species, mainly *L*. *intermedia* and *L*. *whitmani*. However, in peri-urban areas, *L*. *intermedia* is the only vector, and domestic dogs are the reservoirs, a situation that reduces the diversity of the protozoa in this area. This reinforces the idea that *L*. *whitmani* is a connecting vector between the preserved and anthropic environments.

*Lutzomyia lenti* and *L*. *choti* have no association with the occurrence of CL. Despite its occurrence in all regions of Brazil, with a wide distribution and abundance, *L*. *lenti* has not been reported as an important vector in endemic areas of the state of ES. This species has been found in the mid-western region of the country in areas surrounding houses in the state of Mato Grosso do Sul and Goiás, mainly in domestic animal shelters [52–55]. A positive association between this species and cases of CL was observed in the state of Goiás [53]. *Lutzomyia choti* is a species with heterogeneous habitats and is present in both wild environments and areas surrounding houses. However, no records exist of natural infection in this species by *L*. (*V*.) *braziliensis*. In addition, this species has not been observed in the main endemic areas of the state of ES. Brandão-Filho et al. [56] suggested that this species may be associated with the occurrence of CL cases in Pernambuco, northeast Brazil, due to its predominance in the Zona da Mata area of the state.

Regarding the ecological niches constructed in this study, slope was a relevant variable in the occurrence of all species of phlebotomine sand flies. Areas with steep slopes have dimples, which allow a greater variability of water and organic matter accumulation, as well as a reduction in sunlight and wind exposure, thereby creating a greater diversity of habitats; this diversity in habitats contributes to the maintenance of food and shelter conditions for these insects [33].

*Lutzomyia whitmani* is limited to the western region of the state, which has lower precipitation rates, higher elevations, steep slopes, and low temperatures. *Lutzomyia intermedia* is found in warmer areas with lower slope gradients and lower elevations. In addition, this species is less susceptible to temperature and rainfall variations. *Lutzomyia migonei* presents a distribution between *L*. *intermedia* and *L*. *whitmani* and is concentrated in the mid-western region of the state of ES.

Elevation appears to be inversely proportional to the abundance of phlebotomine sand flies [9,11,51,57]. In the current study, *L*. *intermedia* was collected at 1,123 m above sea level but at low density, suggesting the importance of vector density in the occurrence of disease cases in a particular region.

The precipitation and maximum temperature of the warmest month were also relevant to the construction of the models. Temperature and humidity have positive effects on the activity and abundance of phlebotomine sand flies, which depends on the species [47,58]. In the neighbouring state of Rio de Janeiro, *L*. *whitmani* was more abundant in colder and drier months (June, July, and August), whereas *L*. *intermedia* was more abundant in the warmer months of the year (December, January, and February). These data are in agreement with previous studies performed by Souza et al. [47] and Costa et al. [49].

The variables of precipitation seasonality and temperature seasonality were considered important in the occurrence of *L*. *whitmani*, with the latter being more tolerant to variations in temperature and precipitation [18,49].

The temporal mismatch between the species distribution data (1997–2013), the cases data (1978–2013), and climate data (1950–2000) did not significantly affect the modelling results because during the last 50 years, there have been no important environmental changes in peridomiciles in endemic areas of CL in ES. Furthermore, the geographic expansion of the disease occurred from the migration of rural populations to the outskirts of the cities in the 1980s, carrying infected dogs from the western area to east of ES, where vector species were already adapted to the peridomicile [12,14].

A low sandfly frequency in some areas of the state did not influence the species environmental modelling because this method can provide a measure of potential species occurrence in areas not covered by biological surveys and consequently, has become an interesting tool for healthy planning [27].

Maxent presented a strong performance compared with different methods because it performed well and remained stable with respect to the prediction accuracy and the total area predicted, indicating that Maxent can compensate for small species occurrence data sets [59].

The results of the current study indicate that modelling and geoprocessing tools enable a reliable analysis of the association between geo-climatic variables, geographic distribution of vectors, and CL cases in the state of ES. The definition of areas at potential risk for CL transmission allows us to make available relatively accurate information at the regional level that can guide entomological and epidemiological surveillance activities to control the geographic expansion of this endemic disease in vulnerable locations.

## Supporting Information

S1 Table Specimens of phlebotomine sand flies belonging to the 10 most common species collected in the state of Espírito Santo, southeastern Brazil, during the period between 1997 and 2013. (DOCX)

S2 Table Average percentage contribution for five most important environmental variables for each species and CL case. (DOCX)
