# Supplementary material for: Environmental Niche Modelling of Phlebotomine Sand Flies and Cutaneous Leishmaniasis Identifies Lutzomyia intermedia as the Main Vector Species in Southeastern Brazil
Source: PLoS One. 2016 Oct 26;11(10):e0164580. doi: 10.1371/journal.pone.0164580 (PMC5082636; doi:10.1371/journal.pone.0164580)
Supplement: S1 Table — (DOCX) [file pone.0164580.s001.docx]

| **Phlebotomine sand fly species** | **TOTAL** |
| --- | --- |
| *Lutzomyia intermedia* (Lutz and Neiva, 1912) | 171,556 |
| *Lutzomyia longipalpis* (Lutz and Neiva, 1942) | 15,428 |
| *Lutzomyia migonei* (França, 1920) | 11,454 |
| *Lutzomyia choti* (Floch and Abonnenc, 1941) | 10,194 |
| *Lutzomyia lenti* (Mangabeira, 1938) | 9,160 |
| *Lutzomyia whitmani* (Antunes and Coutinho, 1939) | 6,126 |
| *Lutzomyia fischeri* (Pinto, 1926) | 6,058 |
| *Lutzomyia matosi* (Barreto and Zago, 1956) | 4,395 |
| *Lutzomyia hirsutus* (Mangabeira, 1942) | 3,778 |
| *Lutzomyia quinquefer* (Dyar, 1929) | 2,142 |
| Other species | 9,492 |
| **TOTAL** | **249,783** |

**S1 Table. Specimens of phlebotomine sand flies belonging to the 10 most common species collected in the state of Espírito Santo, southeastern Brazil, during the period between 1997 and 2013. File: S1 Table.docx**
