# Supplementary material for: Environmental Niche Modelling of Phlebotomine Sand Flies and Cutaneous Leishmaniasis Identifies Lutzomyia intermedia as the Main Vector Species in Southeastern Brazil
Source: PLoS One. 2016 Oct 26;11(10):e0164580. doi: 10.1371/journal.pone.0164580 (PMC5082636; doi:10.1371/journal.pone.0164580)
Supplement: S2 Table — (DOCX) [file pone.0164580.s002.docx]

**S2 Table. Average percentage contribution for five most important environmental variables for each species and CL case. File: S2 Table.docx.**

| **Phlebotominae species** | **BIO4** | **BIO5** | **BIO11** | **BIO12** | **BIO13** | **BIO14** | **BIO15** | **BIO16** | **BIO17** | **BIO18** | **elevation** | **slope** |
| --- | --- | --- | --- | --- | --- | --- | --- | --- | --- | --- | --- | --- |
| *Lutzomyia choti* | 15.08 | - | - | - | - | - | - | - | 10.31 | 12.70 | 12.35 | 15.61 |
| *Lutzomyia intermedia* | - | 6.33 | - | 8.43 | 13.47 | - | - | - | - | - | 5.63 | 37.85* |
| *Lutzomyia lenti* |  |  | 8.30 |  | 11.24 |  | 29.47* | 8.98 |  |  |  | 11.28 |
| *Lutzomyia migonei* | - | - | - | 6.63 | 14.23 | - | - | 9.66 | - | - | 8.47 | 20.32* |
| *Lutzomyia whitmani* | 26.88* | 3.65 |  |  | 7.62 |  | 7.59 |  |  |  |  | 33.62* |
| CL cases | 27.23* | 5.37 |  | 15.90 |  | 6.64 |  |  |  |  |  | 15.17 |

*****Asterisks indicate statistically significant differences between variables.
